# The impact of a polyphenol-rich supplement on epigenetic and cellular markers of immune age: a pilot clinical study

**DOI:** 10.3389/fnut.2024.1474597

**Published:** 2024-11-18

**Authors:** Austin Perlmutter, Jeffrey S. Bland, Arti Chandra, Sonia S. Malani, Ryan Smith, Tavis L. Mendez, Varun B. Dwaraka

**Affiliations:** ^1^Big Bold Health PBC, Bainbridge Island, WA, United States; ^2^TruDiagnostic Inc., Lexington, KY, United States

**Keywords:** diet and nutrition, epigenetic clocks, aging, immunity, polyphenols, epigenome-wide association study, Tartary buckwheat, food-is-medicine

## Abstract

Age-related alterations in immune function are believed to increase risk for a host of age-related diseases leading to premature death and disability. Programming of the immune system by diet, lifestyle, and environmental factors occurs across the lifespan and influences both makeup and function of the immune system, including immunometabolism. This programming is believed to act in large part through epigenetic modification. Among dietary components that affect this process, polyphenols may play an outsized role. Polyphenols are a widely distributed group of plant nutrients consumed by humans. Certain foods possess distinctive and relatively higher levels of these compounds. One such food is Tartary buckwheat (*fagopyrum tataricum*), an ancient seed historically prized for its health benefits. It is suggested that the specific composition of polyphenols found in foods like Tartary buckwheat may lead to a unique impact on immunometabolic physiological pathways that could be interrogated through epigenetic analyses. The objective of this study was to investigate the epigenetic effects on peripheral immune cells in healthy individuals of a standardized polyphenol concentrate based on naturally occurring nutrients in Tartary buckwheat. This pilot clinical trial tested the effects of consuming 90 days of this concentrate in 50 healthy male (40%) and female (60%) participants aged 18–85 years using epigenetic age clocks and deconvolution methods. Analysis revealed significant intervention-related changes in multiple epigenetic age clocks and immune markers as well as population-wide alterations in gene ontology (GO) pathways related to longevity and immunity. This study provides previously unidentified insights into the immune, longevity and epigenetic effects of consumption of polyphenol-rich plants and generates additional support for health interventions built around historically consumed plants like Tartary buckwheat while offering compelling opportunities for additional research.

**Clinical trial registration:**

ClinicalTrials.gov, Identifier: NCT05234203.

## 1 Introduction

In the last century, in high-income countries, national life expectancy has steadily risen. Concurrent with a longer lifespan, there has been a rapid rise in the prevalence of chronic conditions including cardiovascular diseases, diabetes, cancers, chronic respiratory diseases (e.g., asthma and chronic obstructive pulmonary disease) (1). These represent significant contributors to morbidity and mortality as well as unsustainable increases in healthcare costs. Thus, increasing the number of healthy years lived (i.e., health span) remains a challenge despite the increase in lifespan.

Biological age is described as a local or organismal rate of cellular aging and has emerged as a superior predictor risk for disease, mortality, and morbidity when compared to chronological age. Biological aging is most often measured through evaluation of epigenetic markers, most commonly methylation of the cytosine-guanine (CpG) islands in DNA. Biological aging and epigenetic aging are often used interchangeably. Notably, many epigenetic testing approaches utilize DNA from peripheral immune cells, which allows for simultaneous assessment of immune-related epigenetic markers that change with age. Algorithms using epigenetic methylation data such as GrimAge (2), PhenoAge (3), and DunedinPace (4) have been applied to the determination of biological age of immune cells (5). It has been found that a higher biological age of the immune system represents a significant risk factor for all-cause mortality and reduction in longevity (6–8).

Shifts in immune cell subsets, cytokines, and functions are seen in chronic disease states ranging from cardiovascular disease to dementia. In addition, research has progressively focused on the role of the immune system in the aging process itself. Pharmaceutical and non-pharmaceutical approaches have been proposed as potential avenues for ameliorating the putative damage from immune aging (i.e., immunosenescence). The combined measurement of epigenetic aging metrics and immune-specific measurements may, therefore, provide considerable insights into one’s risk for disease and disability.

A state of relative immunosenescence (age-related changes in immune system makeup and function) may contribute to chronic disease via the generation of senescent cells– cells outside the cell replicative cycle due to stress or other insults (9). While senescence appears to play a key role in tumor suppression, senescent cells can nonetheless remain metabolically active and produce and secrete a wide range of immunologically active molecules, including inflammatory mediators. The development of senescent cells that produce inflammatory mediators has been termed the senescence-associated secretory phenotype (SASP) and is thought to contribute to the higher levels of inflammation that are often seen with advancing chronological and biological age.

Research on interventions to slow the rate of biological aging, including targeting pathways of immunosenescence has primarily been conducted in cell and animal models. Several recent studies have examined the effect of lifestyle and pharmaceutical interventions on biological aging in humans as well. For example, associations between nutritional intervention, or diet and lifestyle, with epigenetic aging have been reported (10, 11). Food-related molecules have been proposed to play an outsized protective role in slowing or potentially reversing epigenetic aging. Among the best-studied health-promoting dietary nutrients are polyphenols, non-caloric plant-derived compounds which have a wide variety of proposed effects in humans. Indeed, consumption of a polyphenol-rich beverage was found to correlate with epigenetic changes in immune cells of dyslipidemic humans in a recent study (12). A recent randomized controlled trial (13) additionally demonstrated epigenetic effects linked to consumption of a polyphenol-enriched Mediterranean diet proposed to have effects on the immune response.

Several lines of research have more explicitly examined slowing or reversing immunosenescence. Here again, polyphenols have been proposed to play a potential role, and in numerous cell and animal studies polyphenols, including quercetin and curcumin, have been implicated in the reversal of various markers of immunosenescence (14, 15).

Polyphenols are a large family of molecules naturally occurring in plants characterized by the presence of multiple phenolic hydroxyl groups providing putative antioxidant potential. Diets high in polyphenols, especially the Mediterranean diet, have been linked to a variety of positive health outcomes. These include lower rates of degenerative diseases like atherosclerosis, as well as improved metabolic function (16, 17). One of the central mechanisms proposed to account for some of these benefits is the effect of polyphenols on immune function and, specifically, in decreasing excessive inflammation. Additionally, it has been postulated that these molecules may exert a positive influence on the gut microbiome by acting as prebiotics, which support healthy microbial communities (18).

While a wide range of polyphenols have been studied in preclinical and clinical trials for their effects on human health, a smaller number have been implicated for potential benefit to both epigenetic age and immunological health. This group includes the molecules quercetin and fisetin. Quercetin occurs naturally in many plants, including red onions, capers, teas, and cruciferous vegetables. Fisetin is found in fruits such as strawberries, kiwis and apples, vegetables like onions and tomatoes, and nuts.

Most research on polyphenols has followed a pharmaceutical-like model, focusing on the isolated effects of individual polyphenols on specific outcomes. However, it is notable that naturally occurring polyphenols are delivered alongside a complex mixture of minerals, vitamins, fiber and other non-caloric plant nutrients (phytochemicals). It is suggested that the benefits of polyphenols may be synergistic when consumed alongside these other plant food components (19, 20). Therefore, naturally occurring combinations of polyphenols and other phytochemicals may be more ideally suited to exert beneficial biological effects than the same molecules consumed in isolation.

### 1.1 Potential mechanism of actions of polyphenols in regulating immunity through epigenetics

Polyphenols are best understood as antioxidants primarily on the merit of *in vitro* studies and chemical structure. It is a widespread belief that the principal health benefit of polyphenol consumption stems from this antioxidant ability. While it is now recognized that oxidative stress (and subsequent antioxidant neutralization) may directly impact epigenetic regulation, including within immune cells, additional mechanisms of polyphenol-induced cellular effects are now being characterized (21). Polyphenols are poorly absorbed in the human GI tract, allowing most dietary polyphenols to reach the large intestine intact, where they are acted upon by the diverse microbes of the gut microbiome (21). Here, polyphenols may be modified by microbial metabolism to generate new metabolites and other bioactive compounds that may impact epigenetic regulation. For example, consumption of polyphenols may induce gut microbes to increase production of short-chain fatty acids [e.g., *β*-hydroxybutyrate (BHB)] which are known epigenetic regulators as well as immune modulators. Polyphenols are additionally acted on by various phase I biotransformation enzymes present in the GI tract prior to absorption, as well as phase II enzymes present in enterocytes and hepatocytes (22). Within circulation, polyphenols and metabolites can bind to immune cells. Polyphenols may modulate epigenetics by way of DNA methylation, histone modification and miRNA expression (23). This has been quantified using epigenetic methylation analysis of immune cell composition with well-respected machine learning algorithms.

### 1.2 Potential metabolic, longevity, and immune pathway effects of polyphenols

Several cellular pathways have been identified as putative drivers of the health impacts of polyphenols within the last decade. From a metabolic perspective, polyphenols have been proposed to act on ceramide production and downstream effects. Ceramides are bioactive lipid species within the sphingolipid class involved in cell-signaling, and their production is known to be regulated by nutritional intervention (24), including preclinical data suggesting a direct impact of polyphenols. Other work suggests an effect of polyphenols on the ubiquitin-proteasome system (UPS), a key cell pathway involved in protein degradation and metabolic function (25). Polyphenols may also exert effects on the Janus kinase-signal transducer and activator of transcription (JAK–STAT) pathway (26), with implications for immunity, metabolism and longevity (27, 28). In addition, the effect of polyphenols on mechanistic target of rapamycin (mTOR) and 5′-Adenosine monophosphate activated protein kinase (AMPK) pathways (29) represents an area of overlap between these three domains.

One challenge in determining the relative contribution of individual pathways to the health-promoting effects of polyphenols is that much of the existing research has focused on the effect of polyphenolic intervention in *in vitro* data looking at specific pathways identified *a priori*, especially antioxidant potential. Thus, a study designed to examine a full spectrum of potential pathway effects of polyphenol and phytochemical interventions in humans presents a unique opportunity to better understand influences of these molecules across disparate biological systems.

### 1.3 Tartary buckwheat

Tartary buckwheat is a buckwheat cultivar used for thousands of years for its medicinal and food properties. It is known to contain phytochemicals that include the polyphenols rutin, quercetin, luteolin and hesperidin, as well as nutrients such as d-chiro-inositol, a cyclic polyol clinically studied for its effects on human metabolism (30–32).

Dietary interventions with Tartary buckwheat have been independently studied and shown to have positive effects on human physiology related to immune function and metabolism (33, 34). The sum of this research suggests that consumption of phytonutrients found in Tartary buckwheat may positively affect human immune function and epigenetic expression, potentially through shared pathways.

With specific regard to the polyphenol content of Tartary buckwheat, it is notable that the seed contains up to 2.42% flavonoid polyphenolic content by weight (35), compared to a significantly lower content in wheat 0.84% (36). Tartary buckwheat is also the most concentrated food source of rutin, a flavonol glycoside polyphenol which demonstrates primarily preclinical efficacy in modulating metabolic and immune pathways (37, 38).

The relatively high and unique polyphenol content of Tartary buckwheat makes it an ideal candidate for further investigation as a health-promoting food, as well as examination of the value of its representative nutrient profile. The supplement used in this trial contains potentially epigenetically- and immunologically- active polyphenols, as well as additional phytochemicals included to better mirror the suite of biologically active phytochemicals naturally occurring in Tartary buckwheat.

We hypothesize that consumption of a polyphenol concentrate based on the makeup of Tartary buckwheat over 90 days will lead to significant changes in the epigenetic methylation patterns and immune cell phenotypes in healthy adults, as measured by epigenetic age clocks and immune markers, potentially contributing to enhanced immune function and longevity-related physiological pathways.

## 2 Materials and methods

### 2.1 Ethical approval and study design

The Institute of Cellular and Regenerative Medicine Institutional Review Board granted approval for all procedures involving humans in this study. This study is registered on ClinicalTrials.gov under the registration number NCT05234203.

### 2.2 Participant recruitment and eligibility

50 generally healthy (defined as the absence of exclusion criteria below) men and women between the ages of 18 and 85 years (inclusive) with body mass index (BMI) <40 kg/m^2^ were enrolled in the study. This age range was selected to capture a diverse range of adult participants while avoiding the potential for outlier variability introduced at extremes of aging, and to capture the population most likely to be taking a nutritional supplement. Participants were required to have an established primary care provider and active health insurance; be able to read, write, and speak English fluently; and be able to comply with the protocol instructions including performing the in-home venous blood draw using a Tasso device. Investors or immediate family members possessing investment in Big Bold Health were excluded from the study.

Women who were pregnant and/or lactating and individuals on jobs requiring night shift work were excluded. Exclusion criteria also included history (prior 2 years) or presence of cancer, except for non-melanoma skin cancer; known history of blood dyscrasias including coagulopathy or use of prescription anticoagulants; diagnosis of a transient ischemic attack (within 6 months); presence of clinically significant acute or unstable cardiovascular or cerebrovascular disease, psychiatric disorder, alcohol or chemical dependence; immune-related conditions (e.g., hepatitis C, HIV, or active infection within the previous 4 weeks) or other illness that in the opinion of the Clinical Investigator would render a participant unsuitable to participate in the study. In addition, those with known allergy to any of the components of the test product, those consuming known prescription immunomodulating products (e.g., oral glucocorticoids, TNF-*α* inhibitors) or concentrated polyphenolic supplements within 1 month the baseline visit. Concentrated polyphenolic substances that were specifically excluded prior to, and during the study were quercetin, rutin, luteolin, epigallocatechin gallate (EGCG), resveratrol, curcumin, fisetin, berberine, soy isoflavones (genistein, daidzein, and glycitein), hesperidin, and ellagic acid.

### 2.3 Study objectives

The primary objective of this exploratory clinical trial was to evaluate the effect of consuming a polyphenol-rich supplement largely based around the phytochemical composition of Tartary buckwheat (HTB Rejuvenate) for 90 days on epigenetically-measured immune age. The duration of the study period was chosen based on previous polyphenol intervention trials (39, 40) and is generally in line with duration of diet-based polyphenol interventional research (41, 42). Additionally, it was determined that this period would allow for multiple rounds of immune cell turnover as the average lifespan of the majority of peripherally-measured immune cells falls in this range (e.g., the majority of circulating immune cells are neutrophils, with a lifespan of less than 5 days, B cell lifespan average is 52 days) (43).

The secondary objective was to assess the effects of HTB Rejuvenate on peripheral leukocyte immune profiles after 90 days, as well as on GO pathways. Tertiary objectives included capture and review of descriptive clinical observations using a General Health Questionnaire (GHQ). Safety was also assessed via reports of adverse events (AEs).

### 2.4 Study product

The study product was a polyphenol-rich supplement (HTB Rejuvenate) that is commercially available and produced under Good Manufacturing Practices (GMP). The HTB Rejuvenate supplement delivered 579 mg of polyphenols per serving (2 capsules), which is within the studied ranges of polyphenol intake consumed through diet (44).

The study product is a concentrated version of naturally occurring phytonutrients in Tartary buckwheat that contains rutin, quercetin, luteolin, hesperidin as well as d-chiro-inositol (Table 1). 2-hydroxybenzylamine (2-HOBA) is an antioxidant phytochemical also isolated from Tartary buckwheat. Hydroxymethylbutyrate (HMB) was also included in the study product as this is a naturally-occurring byproduct of leucine metabolism linked to improvement in age-related metrics including muscle loss (45). Tartary buckwheat is particularly enriched in leucine (46). Test product was consumed at 4 capsules per day, taken as 2 capsules twice per day close to 12 h apart. Specifically, participants were counseled to consume 2 capsules in the morning (between 6 and 10 am) and 2 capsules in the evening (between 6 and 10 pm) with food.

**Table 1:** Composition of HTB Rejuvenate.

Component	Amount per Serving (2 capsules)	Amount per day (4 capsules)
Himalayan Tartary buckwheat (HTB) flour	95 mg	190 mg
D-chiro inositol (DCI/D-chiro-inositol)	150 mg	300 mg
2-hydroxybenzylamine (2-HOBA)	13 mg	26 mg
Hydroxymethylbutyrate (HMB)	69 mg	138 mg
Chlorophyllin	7.5 mg	15 mg
Polyphenols*	579 mg	1,158 mg
Vitamin C	20 mg	40 mg
Calcium	30 mg	60 mg

*Polyphenols delivered in one serving (2 capsules) included 330 mg quercetin, 83 mg rutin, 83 mg hesperidin, and 83 mg luteolin.

### 2.5 Analysis populations

A review of compliance and protocol deviations was conducted prior to data analysis, and those participants whose data is included for analysis populations is summarized in Appendix 1. Based on the completion of the blood sampling for the epigenetic tests, the modified intent to treat (ITT) population, which includes all participants who completed both baseline and final labs, was composed of *n* = 47. One participant withdrew consent at visit 2, and two participants who did not complete the final blood draw were excluded from the ITT population because the outcome required both tests for analysis. In addition to removal of the two individuals who were excluded from the ITT, the per-protocol (PP) population excluded seven other participants, including two for use of excluded medications/supplements, four for low study supplement compliance, and one for both inclusion of an excluded medication/supplement and low compliance. The final analyzed population therefore included 40 people.

### 2.6 Demographics

The demographics obtained during screening/baseline clinical interviews for the ITT and PP populations are provided in Table 2. Participants in the PP population, which was the primary population for the laboratory analyses, were 54 y (SD, 11 y) old with BMI of 24.2 kg/m^2^ (SD, 3.3 kg/m^2^). A total of 14 participants had documented cases of COVID during the study (See Appendix 1).

**Table 2:** Characteristics of participants.

Characteristic	Units	ITT	PP
Population Number	*N*	47	40
Age	y (±SD)	54 (±11)^‡^	54 (±11)^§^
Sex	% Female	60	62.5
Weight (self-report)†	kg	70.3 (±15.7)^‡^	69.2 (±14.7)^§^
Height (self-report)†	cm	166.7 (±11.1)^‡^	166.5 (±11.2)^§^
BMI	kg/m^2^	24.6 (±3.5)	24.2 (±3.3)

BMI, body mass index; cm, centimeter; ITT, intent-to-treat; kg, kilogram; m, meter; *N*, subject number; PP, per protocol; SD, standard deviation; y, years. ^*^The ITT data excluded the two participants who did not complete final lab tests. ^†^Self-reported weight and height data were converted to metric units for reporting purposes. ^‡^The ITT population had no data for 3 participants for age, and 11 participants for weight and height. All participants had reported BMI data. ^§^The PP population had no data for 3 participants for age, and 7 participants for weight and height. All participants had reported BMI data.

### 2.7 Study design

This was a virtual, single-arm open-label prospective pilot clinical trial comparing epigenetic and immune assays in participants prior to and after 90 days of supplementation with the polyphenol-rich HTB Rejuvenate supplement. The study included a screening/baseline visit (Visit 1, Day −15), communication for starting product (Visit 2, Day 0), 24-h check-in after product start (Visit 3, Day 1), and three follow-up visits (Visit 4, Day 30. Visit 5, Day 60; Visit 6, Day 90). In addition, participants were contacted by phone approximately 30 to 90 d after completing the study and when laboratory results were available. Study details are provided in the Study tracking table (Table 3).

**Table 3:** Study tracking table.

	Screening/Baseline	Start product	24-h check-in	Mid-study check-in	Mid-study check-in	End of study
Visit	1	2	3	4	5	6
Day^1^	−15	0	1	30	60	90
TeleVisit	X					X
Phone/email visit^1^		X	X	X	X	
Informed consent^2^	X					
Inclusion/exclusion criteria^3^	X					
Pregnancy verbal screen^3^	X					
Consume test product^4^		X				
Compliance check^5^			X	X	X	X
General Health Questionnaire (GHQ)^6^	X			X	X	X
TruDiagnostic Questionnaire^7^	X					X
Blood collection/Epigenetics Test^8^	X					X
Adverse events (AEs)^9^			X	X	X	X

1 A window of +14 d was allowed between Visit 1 and 2. An email communication was provided prior to Visit 2 instructing participants to start product within 7-d (Visit 2, Day 0). Visits 3, 4, 5, and 7 were conducted by phone call with the Research Associate, and Visits 1 and 6 conducted by TeleVisit with the Clinical Investigator. A window of +24 h was allowed for Visit 3, anchored to product start (Visit 2, Day 0). A window of ±5 days was allowed between Visits 4 and 5, anchored to Visit 2 (Day 0). A window of +10 d was allowed for Visit 6, anchored to Visit 2 (Day 0).

2 HIPAA, Health Insurance Portability and Accountability Act authorization for disclosure of protected health information and Informed Consents. Signed documents authorize the use and disclosure of the subject’s Protected Health Information by the Investigator and by those persons who need that information for the purposes of the study.

3 Inclusion Exclusion Criteria were reviewed at study initiation for eligibility, and at end of study for possible changes and deviations from the study. Inclusion/exclusion review included verbal assessment for pregnancy for all women age < 60 y.

4 Eligible participants were shipped study products after confirmation of eligibility and agreement to participate (Visit 1). Study product was labeled “FOR RESEARCH USE ONLY” and participants were sent an email when confirmation of shipping was obtained. Participants were instructed to begin product consumption within 7 d and schedule the 24-h follow-up call. The first day of product consumption was documented and defined as study Day 0.

5 Participants were verbally queried about compliance at Visits 4, 5, and 6. Participants were provided a paper Study Log to allow for self-monitoring of daily product consumption and documentation of clinical experiences and AEs as an aid.

6 The General Health Questionnaire (GHQ) was sent to participants electronically via the clinical database module prior to Visit 1 (Day −15), Visit 4 (Day 30), Visit 5 (Day 60), and Visit 6 (Day 90), and follow-up queries on completion were made at the subsequent study visits.

7 The TruDiagnostic intake questionnaire, which contains questions on health history, diet and lifestyle, was provided at baseline (Visit 1, Day 0), and a follow-up brief questionnaire was obtained at the final blood sampling, Visit 6, Day 90.

8 Epigenetics testing was conducted at baseline (Visit 1, Day 0) and end of product consumption (Visit 6, Day 90). Blood was obtained in the home by participants using the Tasso device and sent to the TruDiagnostic laboratory, where it was analyzed by epigenetic testing.

9 AEs were obtained by open-ended questioning at Visit 3 (24 h),Visit 4 (30 d), Visit 5 (60 d) and Visit 6 (90 d) and assessed for severity and relationship to product by the Clinical Investigator. The study log included questions for participants to provide daily observations and aid in response to the questions at the subsequent visits.

Participants were pre-screened using an on-line questionnaire and those meeting criteria were provided a detailed study description and the electronic Informed Consent. Interested individuals attended a TeleVisit (Visit 1, Day −15) with the Clinical Investigator, during which it was confirmed that they met all the inclusion and none of the exclusion criteria. Eligible participants who voluntarily signed the Informed Consent were then provided the baseline GHQ electronically and sent the Tasso blood draw device and instructions to obtain an in-home blood sample for the baseline epigenetic testing. Participants were also sent two bottles of study product (120 capsules/bottle) and instructions for starting consumption after baseline data were obtained (Visit 2, Day 0), and scheduling the 24-h call (Visit 3, Day 1). Participants were instructed not to make substantial changes to diet and lifestyle (e.g., dietary and physical activity programs, new supplements) during study period unless explicitly recommended by their treating medical provider.

At Visit 3, participants were asked about adverse events (AEs), product consumption start date was confirmed, and participants were reminded of the study instructions, including not consuming polyphenol supplements and maintaining habitual diet and lifestyle during the study. Mid-study check-in visits were conducted by phone call with the Research Associate at Visit 4 (Day 30) and Visit 5 (Day 60), during which participants were queried for compliance, AEs, and changes to concomitant medications/supplements and habitual diet and lifestyle. In addition, participants were sent the GHQ electronically and queried on completing the questionnaire. Bottles of study product (30 capsules) were sent after Visit 4 and Visit 5.

Prior to the end-of-study visit (Visit 6, Day 90), participants were sent the link to the final GHQ as well as a Tasso blood draw kit. At Visit 6, participants attended a TeleVisit with the Clinical Investigator for the final review, which included study product consumption compliance, completion of the final GHQ, AEs, maintenance of habitual diet and lifestyle, and any changes to concomitant medications and supplements. Confirmation that the final blood was obtained before participants discontinued the study product.

### 2.8 Clinical and anthropometric information

Relevant medical history was reviewed by the Clinical Investigator prior to signing the Informed Consent. Anthropometric information included self-reported height and weight, with the date when the participant last weighed themselves documented, and BMI was calculated. Participants were questioned on medication and supplement use at Visit 1 (Day −15) and asked about changes to medication and supplement use, as well as diet and lifestyle at Visits 4, 5, and 6 (Days 30, 60, and 90).

### 2.9 General health questionnaire (GHQ)

Participants were asked to complete an electronic GHQ (Appendix 2) at baseline and 30-d, 60-d, and 90-d after the start of test product consumption which allowed for collection of subjective data. The GHQ included questions on general health over the previous 4 weeks with answers scored on a 5-point balanced Likert scale. The Likert scale varied randomly from lowest to highest score for questions 3, 4, 7, 9, 11, 12, 13, and 15, and from highest to lowest score for questions 1, 2, 5, 6, 8, 10, and 14 to decrease order-effects. In addition, leading and subjective language were avoided in the question design. The individual health categories included: 1: General Health: questions 1, 2, and 13, score range 3 to 15 points. 2: Gastrointestinal (GI): questions 3 and 4, score range 2 to 10 points. 3: Energy & Mood: questions 5, 10, 11, 12, 14, and 15, score range 6 to 30 points. 4: Allergy & Skin: questions 6, 7, 8, and 9, score range 4 to 20 points.

### 2.10 Compliance

Participants were provided a paper study log (a basic printable tracking document) with the initial product shipment that included a tracking form for documenting the consumption of 2 capsules of study product in the morning and 2 capsules in the evening every day, as well as a section for daily notes for unusual symptoms, illness and reasons for missed product consumption. The study log was not collected but provided to aid participants in answering queries at the relevant visits. Compliance was obtained during Visits 4, 5, and 6 by query and documented as % consumed over the previous 4 weeks from data on the study logs, as verbally provided by participants.

### 2.11 Epigenetic age biomarkers

Blood samples were obtained by participants at baseline and after 90 days of starting the trial using the in-home Tasso device and shipped to the TruDiagnostic laboratory directly. Overall, we analyzed blood samples from 47 adults from an initial sample set of 50 individuals using an Illumina EPICv1 epigenetic panel that analyzed DNA methylation at 850,000 CpG sites prior to and after 90 days of an intervention with a polyphenol-rich supplement designed to mimic major bioactive nutrients found in the plant Tartary buckwheat. Beta values were extracted from IDAT files using the minfi pipeline, and outlier samples were identified using the ENmix R package (47, 48). Final analysis included data from 40 people who successfully completed the study. All analyses were conducted using the R programming environment.

Epigenetic clocks were derived from processed beta values including the OMICmAge clock (49), PhenoAge clock (3) and GrimAge version 1 (2). To ensure high reproducibility, the principal component versions of these clocks were used as outlined by Higgins-Chen et al. (50). The clocks were calculated using a custom R script available on GitHub. Additionally, DunedinPACE was computed using a specific script available on GitHub (4).^1^ Epigenetic assessment was conducted using Wilcoxon-rank sum test performed by evaluators blinded to participant identifying information. For all epigenetic statistical tests, epigenetic measures were converted from raw outputs to epigenetic age acceleration (EAA), which is defined as the residual calculated between the epigenetic age measurement and chronological age. To account for batch effects, the variation calculated from the first three principal components of the control probes were added as fixed effects to the EAA estimation.

Immune assessments were obtained via deconvolution algorithms of the epigenetic data, which quantitatively approximate immune cell subsets including CD4+ T cells, CD8+ T cells, granulocytes, natural killer cells, monocytes, eosinophils, and neutrophils using the Epidish package (51).

### 2.12 Differential methylation analysis

Differential methylation analysis was carried out utilizing processed beta values. The Limma package was employed for three distinct comparisons, identifying differentially methylated loci (DMLs) within the individuals during the trial from baseline to the final timepoint collection (day 90). Multivariate linear models incorporated fixed effects including beadchip, five immune cell percentages (CD4T naive, CD4T memory, CD8T naive, CD8T memory, B naive, B memory, eosinophils, T regulatory, natural killer (NK), and neutrophils (Neu)), the third and fourth three principal components of technical probes, and the Study ID for the individual. Q-Q plots and lambda values were utilized to assess *p*-value inflation or deflation across the methylome (52); the final lambda value of was 0.96 suggesting the Epigenome-Wide Analysis (EWAS) model did not show inflation or deflation. DMLs were identified with an unadjusted *p*-value significance threshold of <0.001. Functional annotation of DMLs was conducted using the GREAT pipeline to identify significant gene ontology terms, as implemented in the rGREAT R package (53).

### 2.13 Safety assessments

Participants were asked about AEs by open-ended questions at Visit 3 (24-h), Visit 4 (Day 30), Visit 5 (Day 60), and Visit 6 (Day 90). The Clinical Investigator reviewed the reports, obtaining follow-up information from participants as needed, and categorized by severity and relationship to study products based upon FDA criteria (detailed in the Study Protocol).

### 2.14 Statistics

As this was a pilot study, sample size was estimated based on previous analyses with epigenetic studies to require a minimum of *n* = 30. A total of 50 participants were enrolled to account for attrition and non-compliance. Populations for statistical analysis were predefined based on inclusion and exclusion criteria (see below). For epigenetic analysis, we used DNA methylation to compare baseline measures against final measures across the entire study population for OMICmAge PhenoAge, GrimAge and DunedinPACE groups. Subgroup and sensitivity analyses were also performed for each of these subpopulations using +1 and − 1 standard deviations from the mean and within/inclusive of 1 standard deviation of the mean and those testing positive to COVID-19 during the study, as accelerated epigenetic aging in the context of COVID-19 has been documented (54). Demographics and analysis of the General Health Questionnaire (GHQ) were conducted in Excel (Microsoft, version 2211) and presented as descriptive statistics. In all epigenetic analyses, the statistical significance was established using a *p* < 0.05 threshold.

## 3 Results

### 3.1 Participant disposition

Fifty-seven individuals were screened on-line and scheduled for an initial visit to assess eligibility. Of these, *n* = 50 individuals attended the on-line screening visit and met eligibility criteria, and *n* = 7 did not meet eligibility criteria, as follows:

- Involved in another trial, *n* = 2
- Lactating, *n* = 1
- On excluded high dose polyphenolic supplements, *n* = 1
- BMI outside accepted range (<40 kg/m^2^), *n* = 1
- Did not show up for screening, *n* = 1
- Did not want name associated with the DNA results in the database *n* = 1

**Figure 1:**
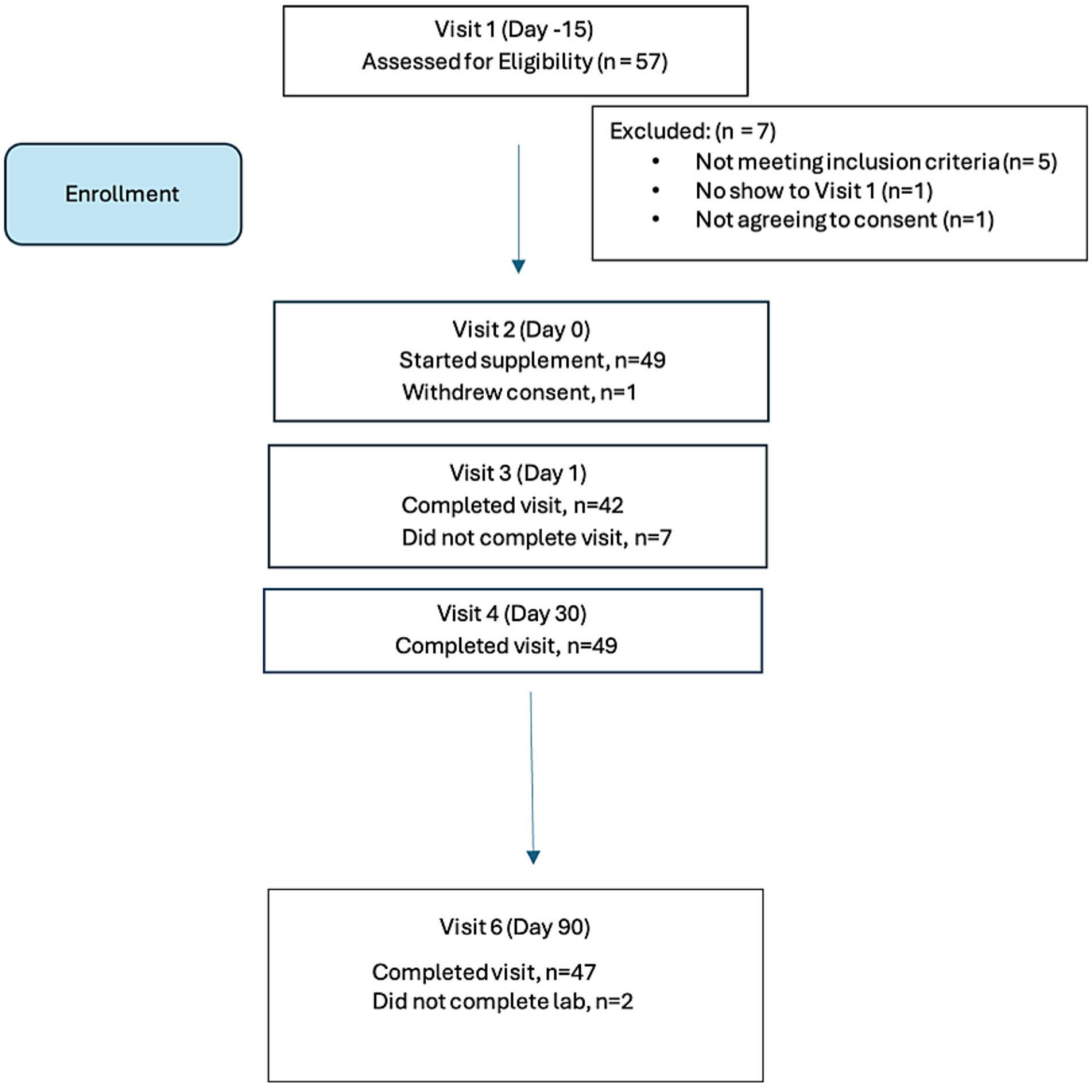
Participant disposition chart.

Of the *n* = 50 enrolled in the study, 47 completed labwork. One participant withdrew consent at Visit 2. In addition, final lab analyses were not obtained from two participants (*n* = 2) and these were considered early terminations (ET) at Visit 6 for purposes of analyses. The participant disposition is summarized in Figure 1.

### 3.2 Epigenome-wide analysis identifies CpGs related with intake of Tartary buckwheat extract

**Figure 2:**
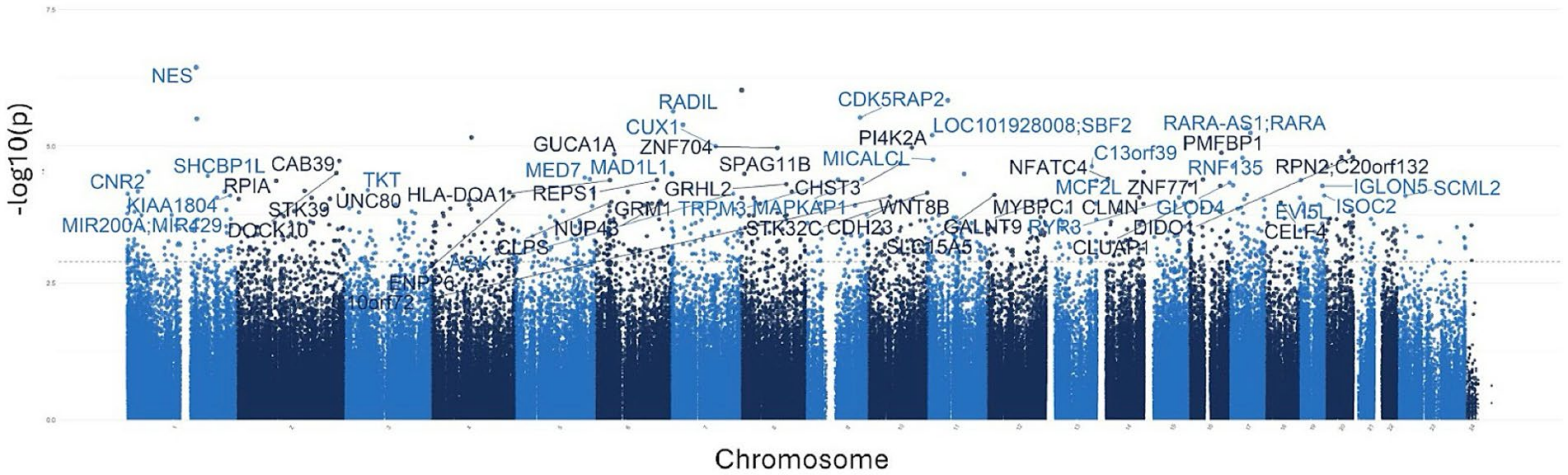
Manhattan plots for the epigenome-wide association study (EWAS). The above plot depicts genes associated with CpG sites identified in the analysis. Each dot on the plot represents a CpG site, with its vertical position corresponding to the negative logarithm (base 10) of the unadjusted *p*-value for DNA methylation association, with a significance threshold set at *p* = 0.001. The x-axis shows genomic positions organized by chromosomes, with color-coded dots indicating specific chromosomes; different shades of blue are used to demarcate separate chromosomes. The prominently peaked dots represent CpG sites that surpass the genome-wide significance threshold, indicating significant associations.

**Figure 3:**
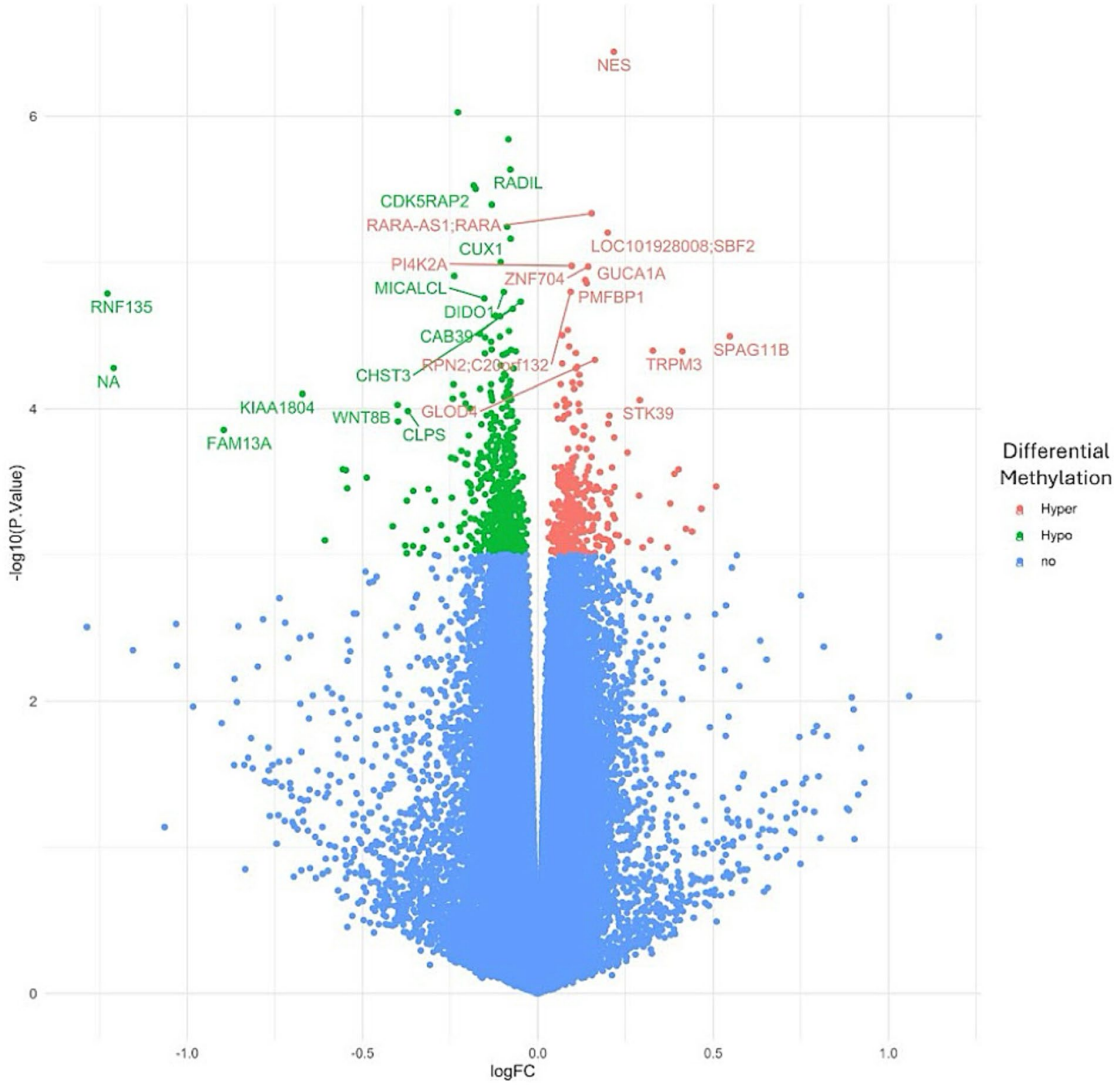
Volcano plot for epigenome-wide association study and enrichment analysis. An epigenome-wide association study and enrichment analysis was conducted to compare pre- and post-intervention data (90 days after starting the study supplement). The Volcano plot illustrates differentially methylated loci (DMLs) identified in the pre- vs. post-intervention comparison. Each dot represents a CpG site, with its vertical position indicating the negative logarithm (base 10) of the unadjusted *p*-value for DNA methylation association. The x-axis shows the relative log fold change (logFC) of the m-values between the two timepoints. Negative values indicate CpGs with decreased methylation among study participants (green), while positive values indicate increased methylation (red).

To investigate the overall epigenetic impact of the standardized polyphenol concentrate upon the cohort, we conducted an epigenome-wide analysis (EWAS) analysis to identify CpGs which showed significant differential methylation between the two visits. To ensure that the EWAS model was not overfit and thus limit false positives, we first tested for inflation by identifying variables which accounted for overfitting. To this end, we estimated the coefficient of overfitting (lambda) at 0.96, which indicated that the EWAS model selected did not show significant overfitting within the data (Supplementary File 2). From the indicated model, we identified 887 Differentially Methylated Loci (DMLs) across the EPIC/850 K data (unadjusted *p*-value <0.001). Among these, 336 CpG sites were hypermethylated at the conclusion of the study (Visit 6), while 551 loci were hypomethylated. The full list of these CpGs is listed in Supplementary File 2. The results of the analysis are further represented in the following Manhattan (Figure 2) and Volcano plots (Figure 3) to allow for visual representation of data. The Manhattan plot is used to identify chromosomal locations of the DMLs and to observe whether DMLs were spatially distributed in clusters or across the genome, whereas the Volcano plot allows for visualization of direction of methylation change (e.g., hyper- vs. hypo-methylation).

### 3.3 Gene ontology pathways

**Figure 4:**
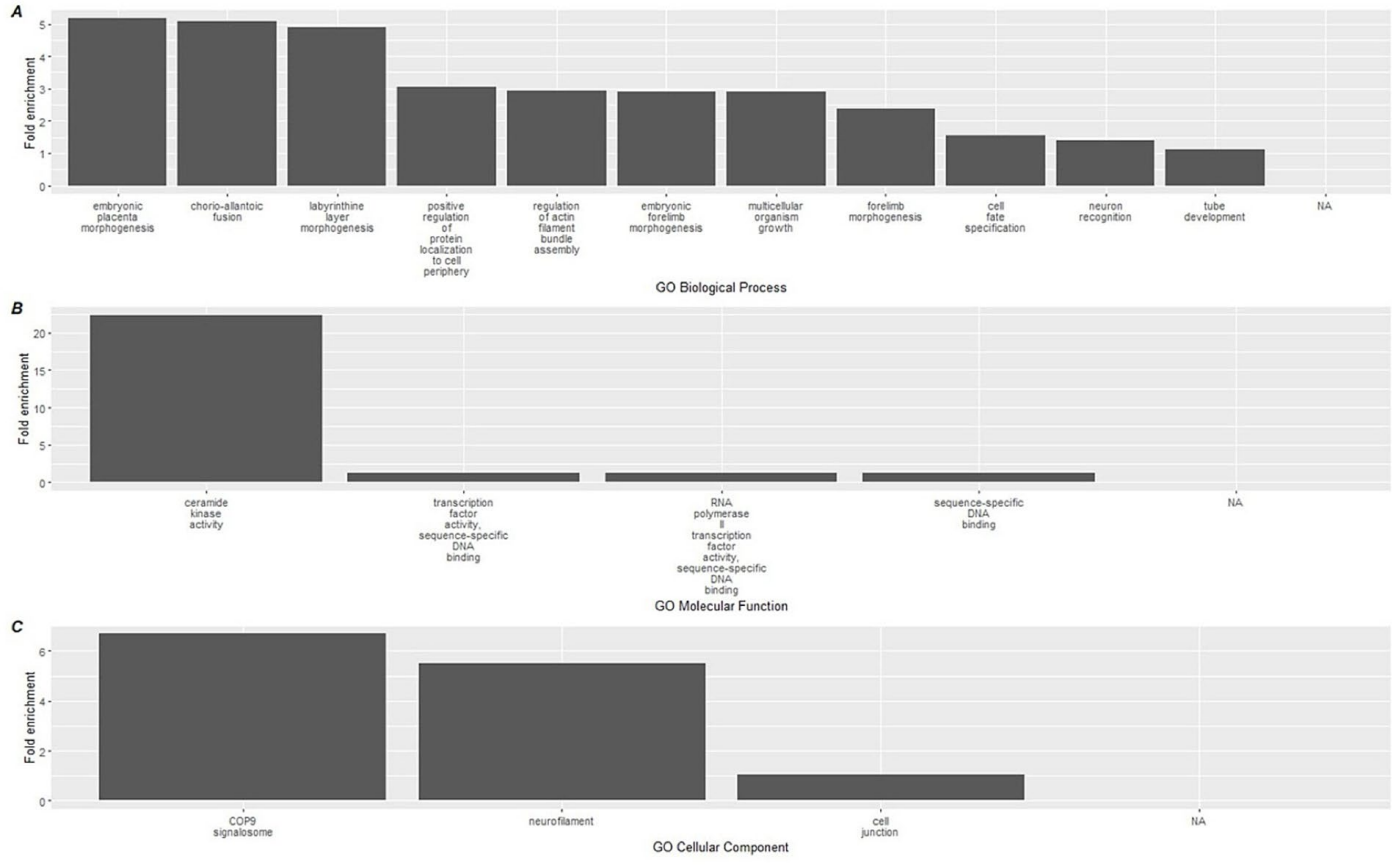
Top 15 significant gene ontology terms associated with hypermethylated DMLs using GREAT. Gene ontology databases are reported for **(A)** GO-BP, **(B)** GO-MF, and **(C)** GO-CC. Biological associations shown are gene ontology (GO) terms for Biological Processes (BP), Molecular Function (MF), and cellular components (CC).

To link the methylation results to biological processes, enrichment analyses using the GREAT software were conducted on CpGs based on the direction of methylation and used to identify gene ontology (GO) pathways. Hypermethylated CpGs at the conclusion of the study were significantly associated with a total of 15 GO-BP (Biological processes) terms, 4 GO-MF (molecular function), and 3 GO-CC (Cellular component) (Supplementary File 3) terms. The top 15 terms for each GO category are shown in Figure 4, which included a diverse group of pathways including ceramide kinase activity, COP9 signalosome activity, labyrinthine layer morphogenesis, and neurofilament activity.

**Figure 5:**
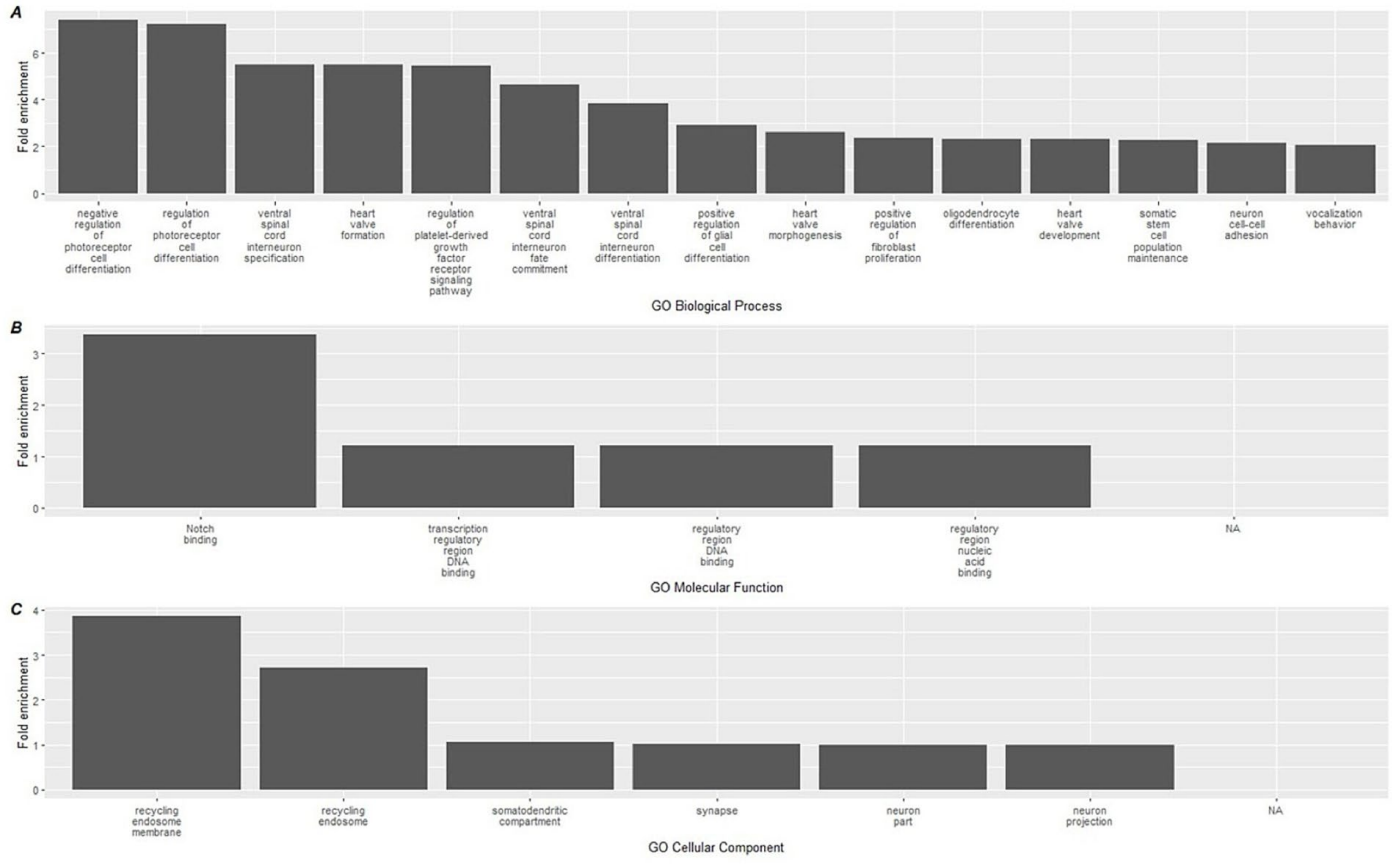
Top 15 significant gene ontology terms associated with hypomethylated DMLs using GREAT. Gene ontology databases are reported for **(A)** GO-BP, **(B)** GO-MF, and **(C)** GO-CC. Biological associations shown are gene ontology (GO) terms for Biological Processes (BP), Molecular Function (MF), and cellular components (CC).

A similar analysis was performed on the hypomethylated DMLs identified in the analysis, which revealed greater significant GO terms compared to the hypermethylated DMLs (Supplementary File 3). Among the hypomethylated DMLs, 124 GO-BP terms, 6 GO-MF terms, and 4 GO-CC terms. The top 15 for each category are reported in Figure 5 which includes the activation of processes that regulate photoreceptor cell differentiation and ventral spinal cord interneuron specification. In addition, we also observed higher enrichment of processes associated with Notch binding.

### 3.4 Comparison to published dietary DML data

**Figure 6:**
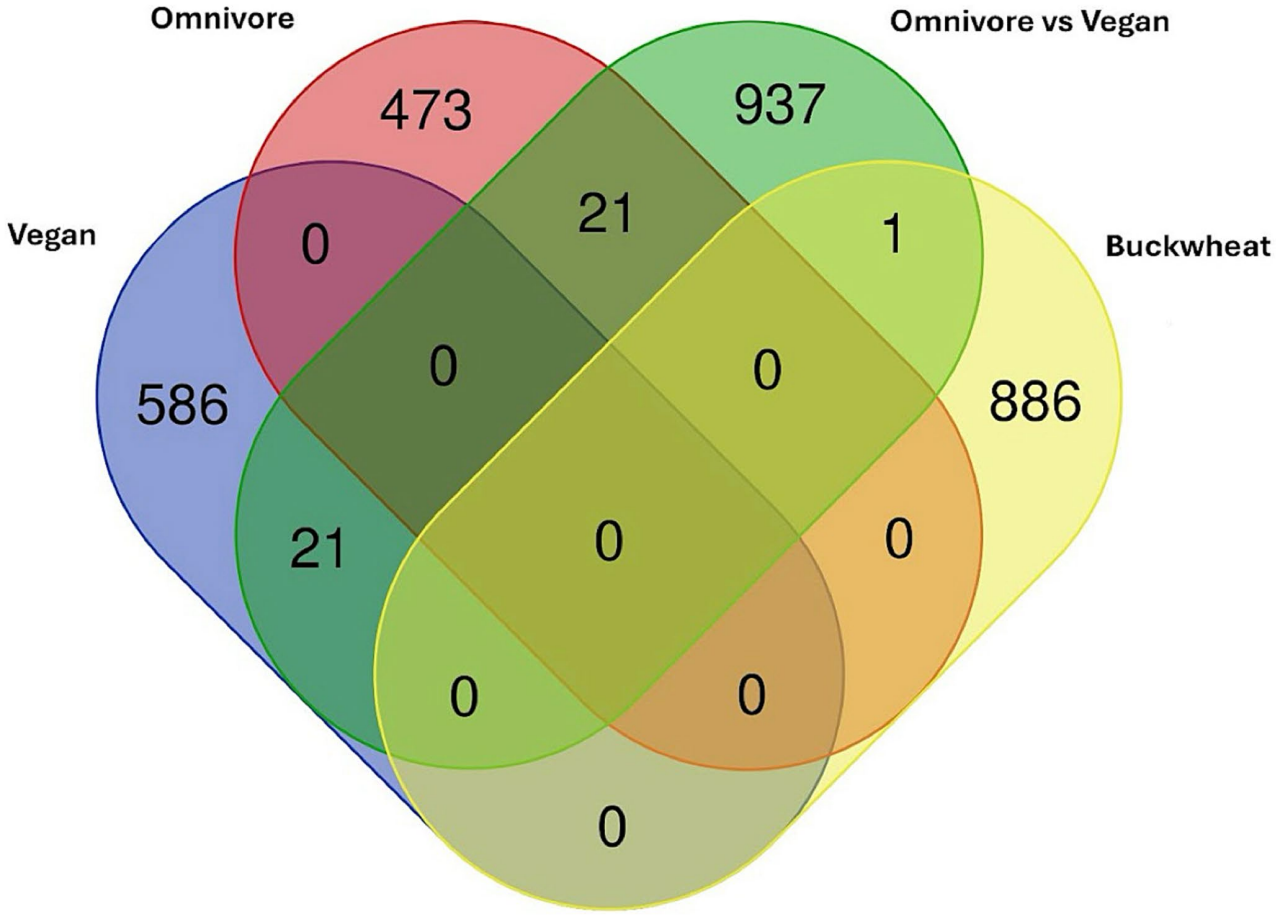
Venn diagram of DMLs identified between the comparisons in (73) plotted against DMLs from the Tartary buckwheat supplement intervention.

To better interrogate whether the above results might represent changes seen in more comprehensive dietary intervention, and whether the study supplement mimicked the effects of a plant-based diet, we compared our data to the results from (73), which examined DMLs across a vegan intervention and omnivore (control) intervention using a Venn diagram of DMLs across the three groups (Figure 6).

The one CpG shared among the current analysis with the Omnivore analysis is cg05093714 (Gene ID: LINC01095) which is significantly higher in the vegan cohort compared to the omnivore cohort at 8 weeks. However, all other CpGs are specific to the Tartary buckwheat cohort identified here. No overlap is observed between the Vegan or Omnivore diet, suggesting different pathways are involved.

### 3.5 Study supplement use impacts changes in epigenetic age

To determine the response to the study supplement on biological age, we quantified and performed analysis on a host of biological age metrics using DNA methylation. Aging clocks used included the second generation multi-omic informed OMICmAge, the third generation DunedinPACE (PACE) and principal component (PC) based second generation PhenoAge and GrimAge clocks. We additionally utilized epigenetic age acceleration (EAA), a marker of the difference between expected rate of aging based on chronological and biological aging. Remarkable findings included: 1. A slowing of epigenetic age acceleration in people with a PCPhenoAge 1SD Higher than the mean (*p* = 0.031) 2. An increase in epigenetic age acceleration in people with a PCGrimAge 1SD Lower than the mean (*p* = 0.031) 3. An increase in epigenetic age acceleration in people with a OMICmAge 1SD lower than the mean (*p* = 0.031). Note that similar *p* values are a result of a wilcoxon-rank sum test which utilizes a rank-based estimate used against smaller *n* values. We stratified sample groups by subsets one standard deviation higher than the mean, one standard deviation lower than the mean, and within/including one standard deviation (−1 to +1) of the mean to better understand the degree to which starting epigenetic state impacts outcome, and as epigenetic changes seen in the context of dietary modification are typically more subtle. The full results of this analysis are represented in Table 4.

**Table 4:** Investigation of epigenetic age measures based on subsets one standard deviation higher than the mean, one standard deviation lower than the mean, and within or equal to one standard deviation (−1 to +1) of the mean for multiple epigenetic aging algorithms.

	Mean – Test 1	SD – Test 1	Mean – Test 2	Mean – SD Test 2	*N*	Wilcoxon (*p*-value)
OMICmAge EAA – 1SD Higher	5.686	1.176	4.185	2.904	7	0.380
OMICmAge – 1SD Higher	61.290	10.649	59.940	11.844	7	---
*OMICmAge EAA* – *1SD Lower*	*−4.969*	*1.037*	*−3.718*	*1.851*	*7*	*0.031*
OMICmAge – 1SD Lower	48.032	5.557	49.404	5.709	7	---
OMICmAge EAA – Within 1SD	−0.291	1.906	−0.031	3.120	26	0.860
OMICmAge – Within 1SD	54.785	7.201	55.268	7.538	26	---
OMICmAge EAA – All	−0.063	3.604	0.062	3.699	40	0.740
OMICmAge – All	54.741	8.439	55.059	8.548	40	---
*PCPhenoAge EAA* – *1SD Higher*	*8.065*	*2.163*	*4.262*	*2.294*	*6*	*0.031*
PCPhenoAge – 1SD Higher	51.004	4.785	47.456	6.345	6	---
PCPhenoAge EAA – 1SD Lower	−9.300	2.886	−5.790	4.221	7	0.078
PCPhenoAge – 1SD Lower	36.754	11.490	40.537	11.347	7	---
PCPhenoAge EAA – Within 1SD	−0.087	2.435	0.166	4.132	27	0.360
PCPhenoAge – Within 1SD	48.759	10.207	49.251	10.601	27	---
PCPhenoAge EAA – All	−0.476	5.580	−0.262	4.854	40	0.690
PCPhenoAge – All	46.995	10.778	47.457	10.522	40	---
PCGrimAge EAA – 1SD Higher	2.960	0.822	2.177	1.455	8	0.200
PCGrimAge – 1SD Higher	63.209	11.339	62.561	11.717	8	---
*PCGrimAge EAA* – *1SD Lower*	*−3.759*	*0.554*	*−1.333*	*0.536*	*6*	*0.031*
PCGrimAge – 1SD Lower	57.527	12.238	60.078	12.357	6	---
PCGrimAge EAA – Within 1SD	−0.407	1.165	−0.336	2.166	26	0.670
PCGrimAge – Within 1SD	61.605	7.425	61.855	7.860	26	---
PCGrimAge EAA – All	−0.236	2.248	0.017	2.178	40	0.320
PCGrimAge – All	61.314	8.979	61.730	9.187	40	---
PACE – 1SD Higher	1.007	0.050	0.976	0.071	8	0.250
PACE – 1SD Lower	0.710	0.033	0.699	0.090	7	0.690
PACE – Within 1SD	0.841	0.050	0.866	0.079	25	0.110
PACE – All	0.851	0.104	0.859	0.116	40	0.610

Italicized terms meet statistical significance at a *p*-value of 0.05 or less.

**Figure 7:**
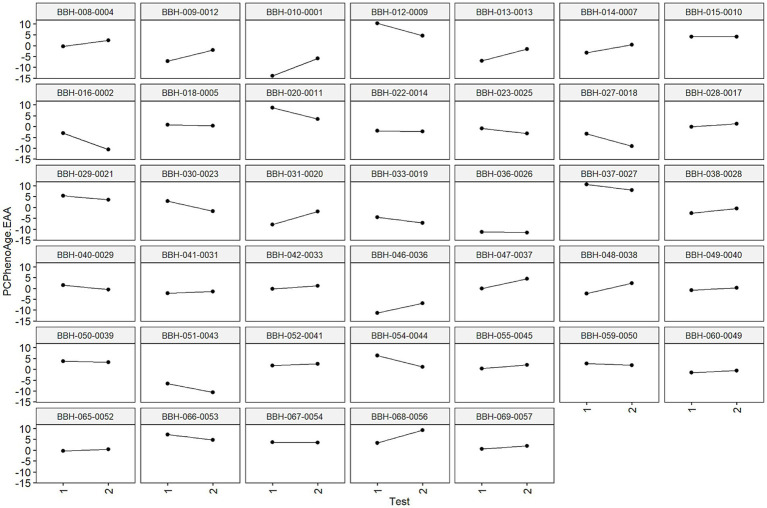
Visual representation of individual epigenetic age accelerations (PCPhenoAge used as example). Each graph is faceted by each individual’s de-identified ID provided during the trial. Individual slopes calculated as changes in signify changes in epigenetic age metrics during this window (positive slopes represent increased epigenetic age acceleration, negative slopes represent decreased epigenetic age acceleration). These results may speak to individual differences in epigenetic sensitivity to environmental inputs, in this case dietary modification.

It is important to note the heterogeneity of individual results measured using age-related algorithms across the 90-day study period. An individual’s epigenetic response to environmental factors and interventions may be significantly influenced by their pre-existing epigenetic status as well as a host of other factors known to impact epigenetic expression (e.g., gut microbiome composition, immune cell makeup, baseline exercise and dietary regimen). To this end, we’d expect diversity in epigenetic age-related outcomes across participants. This can be visualized in Figure 7 using data on the 40 study participants included in the final analysis. Positive slopes represent increased epigenetic age acceleration and negative slopes represent decreased epigenetic age acceleration across the 90-day study period.

### 3.6 Deconvoluted immune cell analysis

Alterations in immune cell makeup and function have been studied in references to both dietary change and specific nutrient augmentation. We used deconvolution methods to determine immune cell population changes over the duration of the study period to explore the effects of study supplementation on immune cell parameters across different biological age algorithms and within subsets of study participants. The PhenoAge algorithm has been independently validated to correlate with multiple markers of immunosenescence which are serologically determined (3). These include populations of T cells, B cells and granulocytes. To this end, we chose to apply the deconvolution methods to PCPhenoAge subgroupings. These can be reviewed in Table 5.

**Table 5:** Representation of changes in deconvoluted immune cell subsets using epigenetic age accelerations for PCPhenoAge.

	Week 0	Week 8	Wilcoxon – *p*-value	Clock comparison
CD4Tmem	0.096	0.095	0.934	PCPhenoAge – Within
CD8Tmem	0.053	0.052	0.427	PCPhenoAge – Within
CD4Tnv	0.073	0.077	0.117	PCPhenoAge – Within
CD8Tnv	0.034	0.032	0.178	PCPhenoAge – Within
Bmem	0.020	0.019	0.645	PCPhenoAge – Within
Bnv	0.042	0.040	0.470	PCPhenoAge – Within
Treg	0.008	0.009	0.427	PCPhenoAge – Within
Baso	0.019	0.018	0.594	PCPhenoAge – Within
Eos	0.012	0.009	0.786	PCPhenoAge – Within
**NK**	**0.060**	**0.052**	**0.046**	**PCPhenoAge – Within**
Neu	0.521	0.540	0.441	PCPhenoAge – Within
Mono	0.063	0.057	0.220	PCPhenoAge – Within
CD4Tmem	0.105	0.087	0.156	PCPhenoAge – 1SD Lower
CD8Tmem	0.059	0.045	0.219	PCPhenoAge – 1SD Lower
CD4Tnv	0.143	0.118	0.109	PCPhenoAge – 1SD Lower
CD8Tnv	0.055	0.039	0.078	PCPhenoAge – 1SD Lower
Bmem	0.019	0.017	0.297	PCPhenoAge – 1SD Lower
**Bnv**	**0.067**	**0.055**	**0.031**	**PCPhenoAge – 1SD Lower**
Treg	0.013	0.011	0.469	PCPhenoAge – 1SD Lower
Baso	0.018	0.014	0.016	PCPhenoAge – 1SD Lower
Eos	0.007	0.003	0.402	PCPhenoAge – 1SD Lower
NK	0.063	0.057	0.469	PCPhenoAge – 1SD Lower
Neu	0.398	0.512	0.109	PCPhenoAge – 1SD Lower
Mono	0.052	0.042	0.375	PCPhenoAge – 1SD Lower
**CD4Tmem**	**0.071**	**0.107**	**0.031**	**PCPhenoAge – 1SD Higher**
**CD8Tmem**	**0.037**	**0.055**	**0.031**	**PCPhenoAge – 1SD Higher**
CD4Tnv	0.044	0.063	0.063	PCPhenoAge – 1SD Higher
CD8Tnv	0.034	0.042	0.156	PCPhenoAge – 1SD Higher
Bmem	0.014	0.020	0.031	PCPhenoAge – 1SD Higher
Bnv	0.032	0.039	0.156	PCPhenoAge – 1SD Higher
Treg	0.007	0.005	0.438	PCPhenoAge – 1SD Higher
Baso	0.014	0.019	0.156	PCPhenoAge – 1SD Higher
Eos	0.002	0.007	0.201	PCPhenoAge – 1SD Higher
NK	0.029	0.043	0.063	PCPhenoAge – 1SD Higher
Neu	0.654	0.548	0.063	PCPhenoAge – 1SD Higher
Mono	0.061	0.051	0.563	PCPhenoAge – 1SD Higher

Bolded rows indicate a significant *p* value.

### 3.7 Analysis of GHQ results

The GHQ data were reviewed for completeness prior to analysis. Results were obtained for all 49 participants for the baseline and 90-d questionnaires. However, 10 datapoints were missing from the 30-d questionnaires and five datapoints were missing in the 60-d data set. Given this was the first virtual study with this questionnaire, learnings on how easy it was to obtain the data, as well as whether participants were consistent in reporting on the questionnaire was of use. As shown in Table 6, the time between questionnaires ranged widely.

**Table 6:** Time between GHQ responses.

GHQ Timepoint	# Participant responses	Days from baseline GHQ			
		Theoretical	Median	Min	Max
Baseline	49	n/a	n/a	n/a	n/a
Day 30	39	30	37	−4	75
Day 30*	37	30	39	18	75
Day 60	44	60	73	53	111
Day 90	49	90	101	67	138

GHQ, General Health Questionnaire; n/a, not applicable. *Two participants indicated taking the 30-d GHQ before consuming the product, resulting in minus days. The data in this line shows median min and max days from baseline when these individuals were removed from the analysis.

In addition, two participants indicated taking the baseline GHQ after the start of supplement which meant the true baselines were not available for these individuals. Therefore, their data were removed from further analysis of the GHQ. Two participants began supplements that could directly affect the data and were also removed. Therefore, a total of *N* = 36 responses were reviewed for the GHQ. Summary statistics from these respondents are provided in Appendix 3.

The GHQ was provided to participants to gauge for the potential presence of changes in subjective health-related symptoms and metrics. However, these metrics were largely unchanged when analyzing the population across the study period. In general, the population began the study in very good health by self-report. Overall, few changes were noted in any category, however, the population began with middle to high ratings in all areas of health. Therefore, changes in health were not likely to be observed in this population.

## 4 Discussion

### 4.1 General discussion

Dietary interventions for modulating health have been well-documented for millennia. In the last hundred years, we have increasingly understood that food represents a complex mixture of caloric and non-caloric components capable of impacting physiology through myriad pathways. In this context, polyphenols have emerged as potential modifiers of human health, including immune health and longevity. However, beyond antioxidant effects, the specifics of *how* polyphenols and foods containing high levels of polyphenols and other phytochemicals impact health have remained relatively poorly interrogated.

Recent publications suggest the role of a polyphenol-rich diet in modulating epigenetics through direct gene methylation and through differential transcription of genes related to epigenetic regulation (13). Individual studies have especially highlighted the role of flavonoid polyphenols in their action on epigenetics. For example, a 2018 trial in cells taken from type 2 diabetic patients found significant changes in histone acetylation after six months of supplementation with resveratrol (74), while a 2013 study found multiple epigenetic alterations associated with cocoa supplementation for two weeks in participants with metabolic dysfunction (75). With roughly 88% of American adults possessing at least one characteristic of metabolic dysfunction, this nevertheless maintains relevance for most of the population (76). Coffee consumption (a top source of dietary polyphenols) has additionally been associated with alterations in methylation across CpG sites (77), which interestingly was only found in peripheral immune cells (as opposed to saliva-derived cellular epigenetic analysis).

In this 90-day study, we provide some of the first clinical evidence suggesting that combinations of polyphenols and phytonutrients occurring naturally in Tartary buckwheat may have multiple effects on markers of longevity and immune system makeup.

By comparing participant’s epigenetic analyses before and after the 90-day intervention period, we were able to observe statistically significant changes in rate of aging as measured by the PCPhenoAge and PCGrimmAge and OmicAge algorithms in subgroups experiencing higher and lower rates of aging, respectively. This suggests the potential for the combination of nutrients administered to exert an effect on aging parameters in immune cells.

### 4.2 Discussion of results

In analysis of immune cell subtypes using deconvolution methods, we hypothesized the potential for immunosenescence-related immune cell changes. Therefore, we ran the deconvolution methods against subset-specific data in Table 5 above for the PCPhenoAge evaluation. Notable here were significant increases in CD4 T memory cells (PCPhenoAge – 1SD Higher) and CD8 T memory cells (PCPhenoAge – 1SD Higher), significant decreases in B naive cells (PCPhenoAge – 1SD Lower) and significant decreases in Natural Killer cells (PCPhenoAge – Within 1 SD). While the PCPhenoAge 1SD higher population saw a decrease in speed of epigenetic age progression, this group also demonstrated an increase in CD4T and CD8T memory cells. Conversely, those starting the study at a lower overall epigenetic age score saw a decrease in naive B cells. These alterations in adaptive immune cells speak to potential effects on immune phenotypes over a 90-day interventional window. Additional and more comprehensive profiling of immune cell and cytokine alterations linked to Tartary buckwheat nutrient intake may better characterize the immune effects of dietary consumption of this seed, including on immunosenescence-related pathways.

Using genome-wide EWAS analysis comparing blood samples from the start and end of the study period, we identified 887 differentially methylated CPG sites at a *p* value of <0.001 with 336 hypermethylated and 551 hypomethylated sites controlled for potential overfitting. These differentially methylated sites were then analyzed using the GREAT software to identify GO pathways.

When we mapped CpG methylation against known biological processes using the GREAT software, a total of 22 pathways were linked to hypermethylated CpGs, while 134 were linked to hypomethylated CpGs. On review of these processes, the most highly enriched changes in biological pathways occurred within the hypermethylated CpG sites, where the largest fold enrichment (22x) was linked to ceramide kinase activity. We additionally found a 6.7-fold enhancement in COP9 activity. Among the hypomethylated biological processes, the largest enrichment changes were seen with negative and positive regulation of photoreceptor cell differentiation (7.41 and 7.21-fold, respectively). While of lower overall effect magnitude, it is also notable in the context of immunity that positive regulation of glial cell maturation was among the top 10 most pronounced findings, at a 2.9x fold-enrichment. These relatively large effects, in contrast with the immune cell deconvolution results, suggest that the more pronounced impact of the dietary supplementation occurred on upstream immune-related biological pathways measurable through epigenetics. The complete list of GREAT software identified GO pathways can be found in Supplementary File 3.

### 4.3 Review of hypermethylated pathways

The single largest fold enrichment pathway in the hypermethylated analysis (and across all GO pathways) was related to ceramide kinase regulation. Ceramides, bioactive lipids found in plasma membranes, regulate numerous cellular processes, such as cell cycle and immunity, by acting as second messengers (55, 56). Elevated circulating ceramide levels are linked to cardiometabolic and immunological dysfunction, often triggered by dietary factors. For instance, palmitic acid raises ceramide levels, whereas polyunsaturated fats lower them (57).

Ceramide kinase (CERK) converts ceramide to ceramide-1-phosphate (C1P), an important regulator of inflammation and immune responses, with context-dependent pro- or anti-inflammatory effects (58). CERK promotes mast cell degranulation (59) and contributes to cellular senescence, a key factor in aging and inflammation. Inhibiting CERK reduces senescent cell burden, while C1P enhances cell survival (60, 78).

Polyphenols like rutin have been shown to modulate the ceramide pathway, reducing ceramide levels in preclinical models (61). Additionally, adherence to the Mediterranean diet is associated with lower ceramide levels in humans (62). A network pharmacology study identified RAF1, a protein involved in cellular proliferation, as a target of Tartary buckwheat, with ceramide-linked phosphorylation playing a role (63, 64). These findings suggest that Tartary buckwheat may influence immune function, longevity, and epigenetic outcomes by inhibiting the ceramide kinase pathway.

Regarding COP9, this is a conserved protein complex composed of 9 subunits found across eukaryotic cells, and well described in plants and animals. In plants, the COP9 signalosome regulates gene expression and resilience to abiotic stress. COP9 is an established cell-cycle regulator through impacts on ubiquitination and transcriptional modification (65). Genes associated with the COP9 signalosome are linked to regulation of senescence (66). The COP9 signalosome has more recently been implicated in the modulation of neuroinflammation including effects on microglial cells and appears to play a key role in maturation of the autophagosome (67, 68) It has previously been shown that curcumin polyphenols are capable of impacting COP9 (69).

### 4.4 Review of hypomethylated pathways

A relatively higher number of hypomethylated GO pathways were identified by the GREAT software. These diverse biological pathways share a common theme of regulating cell differentiation and development. For example, some of the highest fold change effects occurred in processes governing photoreceptor cell differentiation, ventral spinal cord interneuron specification, oligodendrocyte and generally glial cell differentiation, highlighting mechanisms of neural development and functionality. Of note, polyphenols have been shown in preclinical work to have effects on both neurons and on glial cells (70, 71). Additionally, pathways such as heart valve formation, morphogenesis, and prostate gland epithelium morphogenesis emphasize the precise orchestration of cellular proliferation and specialization necessary for organogenesis. Regulation of signaling pathways, like those involving platelet-derived growth factors or the Wnt signaling pathway, further underscores the delicate balance of activation and inhibition required to maintain tissue homeostasis, control cell fate, and prevent aberrant growth or differentiation. Collectively, these pathways underscore influences on the interplay of various signals that guide the formation, specialization, and maintenance of cells across different biological systems.

### 4.5 Changes across diverse immune and longevity pathways suggest specific role for Tartary buckwheat in “Food is Medicine” discussion

Suboptimal diet has been clearly established as a major risk factor for global death and disability (72). However, recommendations around dietary modification are largely generalized (e.g., eat more fruits, vegetables and whole grains). While this may help at a population level in preventing disease, there has also been a call to understand whether certain foods and nutrients (beyond total calories, macro and micronutrients) may serve to provide an outsized benefit in the health of the individual. As immune-mediated or related conditions are among the most common chronic diseases, dietary interventions targeting immune dysfunction represent an important part of the conversation of “food is medicine.”

Data from this pilot study helps provide a more nuanced understanding of how combinations of naturally occurring phytochemicals might induce beneficial effects on humans through activation of biological pathways involved in immune modulation and immune aging. Compared to pharmaceuticals operating with high potency effects on receptors, the biological impact of naturally occurring nutrients like polyphenols at levels similar to what has typically been found in diets of long-lived populations around the world is expected to be more gradual in onset and less pronounced in effect. Here, we provide preliminary data demonstrating the impact of select phytochemicals from Tartary buckwheat in effecting changes across biological pathways involved in health and disease.

### 4.6 Limitations

As this was a pilot study and did not include a placebo arm, it is possible that some of the effect seen over the 90-day period was a representation of background epigenetic change due to environmental variables. Additionally, this trial did not study dose associations with epigenetics as only one dose of the study supplement was delivered over the course of the study period. While participants were asked not to make any overt changes to their dietary plan or to start any new supplements that might influence the effect of the study supplement, this was a free-living study and therefore participants were not closely monitored. Similarly, variability in lifestyle and related factor (e.g., sleep quality, exercise frequency, stress) were not controlled for in this study and therefore may have contributed to some of the epigenetic changes observed. Finally, this was a relatively small study, with 40 participants included in the final analysis.

### 4.7 Future research considerations

As this was a pilot study, only one dose of polyphenol-rich supplement was studied. However, a future investigation would be benefited by testing the relative immune and epigenetic associations seen with higher and lower doses of polyphenols, as well as testing use of these molecules over a shorter and longer timespan. Additionally, it is known that the host microbiome may play a significant role in polyphenol metabolism, and that it may modulate the influence of polyphenols on the gastrointestinal immune system. To this end, future research that examines correlations between the microbiome and the effects of polyphenol supplementation on immune and epigenetic parameters is worthy of consideration. While this study benefited from deconvolution algorithms that allowed us to examine the effects of intervention across various immune cell subtypes, a deeper immunophenotyping using flow cytometry techniques could allow for interrogation of whether polyphenol-related effects were specific to leukocyte subsets and resultant cytokine production. This degree of precision would aid future efforts to tailor nutritional intervention to individuals.

## 5 Conclusion

In this study, we present novel data indicating the potential for phytochemical nutrients found within Tartary buckwheat to act on multiple metrics of epigenetic immune age, immune cells and related cellular pathways. The polyphenol-rich supplement designed around key bioactive nutrients naturally occurring in Tartary buckwheat appeared to directionally influence CpG methylation patterns in subsets of participants with higher and lower rates of biological aging as measured by the PCPhenoAge, PCGrimAge and OmicAge aging algorithms. These changes were correlated with changes in peripheral immune cell patterns as measured by the PCPhenoAge aging algorithm which is known to be sensitive to changes associated with immunosenescence. Changes in GO pathways additionally suggest the potential for effects on multiple immune and cellular regulatory mechanisms, especially those related to ceramide kinase. These results suggest that polyphenols and associated bioactive nutrients naturally occurring in Tartary buckwheat may significantly influence epigenetic age measurements that may be driven by or reflected in changes in the immune system and associated pathways.

In the context of the larger intersections between the fields of nutrition and healthy aging, this research provides additional support for uncovering pathway-specific and precision nutritional interventions. The detailed analysis generated by epigenetic analysis permits a more comprehensive demonstration of which biological systems may be most modulated by individual or combinations of nutrients naturally occurring in food or supplements, with in this case, a specific focus on Tartary buckwheat. Moving forward, a deeper understanding of potential nutritional targets within an individual’s epigenetic analysis prior to intervention will aid researchers and clinicians in accurately matching nutritional needs with dietary plans and diet adjuncts including supplements.

## Data Availability

The data that support the findings of this study are not publicly available due to protection of patient data in accordance to maintaining HIPAA compliance. However, the corresponding authors can provide the data upon reasonable request after signing a Data Use Agreement. Once signed, corresponding authors will provide a protected link to AWS cloud storage to download raw data.

## Footnotes

^1^https://github.com/danbelsky/DunedinPACE
