# Supplementary material for: The impact of a polyphenol-rich supplement on epigenetic and cellular markers of immune age: a pilot clinical study
Source: Front Nutr. 2024 Nov 18;11:1474597. doi: 10.3389/fnut.2024.1474597 (PMC11612904; doi:10.3389/fnut.2024.1474597)
Supplement: Supplementary file 1 [file Data_Sheet_1.PDF]

Supplementary File 1

Contents: Appendix 1-3

Appendix 1: Population Selection Summary Chart

**Study ID**	**COVID Status**	**Include in ITT**	**Include in PP**	**Rationale**
BBH-008-0004	No	YES	YES	Added a supplement that could impact questionnaire data
BBH-009-0012	No	YES	YES	Added a supplement that could impact questionnaire data
BBH-010-0001	No	YES	YES	
BBH-012-0009	No	YES	YES	
BBH-013-0013	No	YES	YES	
BBH-014-0007	No	YES	YES	
BBH-015-0010	YES	YES	YES	
BBH-016-0002	YES	YES	YES	
BBH-017-0003	No	YES	NO	Added a curcumin supplement
BBH-018-0005	No	YES	YES	
BBH-019-0008	No	YES	NO	Low HTB compliance
BBH-020-0011	No	YES	YES	
BBH-022-0014	No	YES	YES	
BBH-023-0025	No	YES	YES	
BBH-024-0016	No	NO	NO	Did not complete, no 2nd lab test
BBH-027-0018	No	YES	YES	
BBH-028-0017	No	YES	YES	

BBH-029-0 021	No	YES	YES	
BBH-030-0 023	No	YES	YES	
BBH-031-0 020	YES	YES	YES	
BBH-033-0 019	YES	YES	YES	
BBH-036-0 026	YES	YES	YES	
BBH-037-0 027	No	YES	YES	
BBH-038-0 028	YES	YES	YES	
BBH-039-0 030	No	YES	NO	Low HTB compliance
BBH-040-0 029	No	YES	YES	
BBH-041-0 031	YES	YES	YES	
BBH-042-0 033	No	YES	YES	
BBH-043-0 032	No	NO	NO	Did not complete, no 2nd lab test
BBH-044-0 034	No	YES	NO	Low HTB compliance
BBH-046-0 036	No	YES	YES	
BBH-047-0 037	YES	YES	YES	
BBH-048-0 038	YES	YES	YES	
BBH-049-0 040	YES	YES	YES	
BBH-050-0 039	YES	YES	YES	
BBH-051-0 043	No	YES	YES	
BBH-052-0 041	YES	YES	YES	
BBH-053-0 042	YES	YES	NO	Low HTB Compliance
BBH-054-0 044	No	YES	YES	
BBH-055-0 045	No	YES	YES	

BBH-057-0 047	No	YES	NO	Post-menopausal and started estriol
BBH-058-0 048	No	YES	NO	Added supplements and low HTB compliance
BBH-059-0 050	No	YES	YES	
BBH-060-0 049	No	YES	YES	
BBH-065-0 052	No	YES	YES	
BBH-066-0 053	No	YES	YES	
BBH-067-0 054	YES	YES	YES	
BBH-068-0 056	No	YES	YES	
BBH-069-0 057	No	YES	YES	

Appendix 2: GHQ Questionnaire

Instructions: These questions are about how you feel and how things have been with you **during the past 4 weeks.** For each question, please give the one answer that come closest to the way you have been feeling. Choose only one option for each question.

1.Over the past 4 weeks, would you say your health is:

- ○ Excellent
- ○ Very Good
- ○ Good
- ○ Fair
- ○ Poor

2. Considering how you were feeling prior to the past month, would you say that your overall health has changed?

- ○ Yes – Much better over the last 4 weeks
- ○ Yes – Somewhat better over the last 4 weeks
- ○ No – About the same as before
- ○ Yes – Somewhat worse over the last 4 weeks
- ○ Yes – Much worse over the last 4 weeks

3. Considering gastrointestinal effects, such as reflux, heartburn, stomach rumbling, bloating, intestinal pain and discomfort, and flatulence, please comment on your experience over the past 4 weeks.
    - Much worse (higher number) and/or more intense symptoms than usual
    - Somewhat worse than usual (number and/or intensity)
    - No change in symptoms compared to usual
    - Somewhat fewer and/or less intense symptoms than usual
    - Much less in number and/or less intensity than usual

4. Have you had changes to your bowel habits (frequency of going to the bathroom, comfort of bowel movement, form of stool (e.g., loose and watery or very hard) over the past 4 weeks?
    - Much worse than usual
    - Somewhat worse than usual
    - About the same as usual
    - Somewhat better than usual
    - Much better than usual

5. Over the past 4 weeks, have you experienced brain fog?
    - Not at all
    - Infrequently
    - Occasionally
    - Often
    - Very often

6. Considering the health of your skin over the last 4 weeks, have you experienced rashes, felt your skin is itchy or otherwise unhealthy?
    - Not at all
    - Infrequently
    - Occasionally
    - Often
    - Very often

7. If you have skin symptoms, were these:
    - Much worse than usual

- Somewhat worse than usual
- About the same as usual
- Somewhat better than usual
- Much better than usual

8. Over the past 4 weeks, other than an actual cold or illness, have you experienced Upper Respiratory Tract (URT) symptoms, such as runny nose, congestion or sneezing?
    - Not at all
    - Infrequently
    - Occasionally
    - Often
    - Very often

9. Over the past 4 weeks, would you say your Upper Respiratory Tract (URT) symptoms were?
    - Much worse than usual
    - Somewhat worse than usual
    - About the same as usual
    - Somewhat better than usual
    - Much better than usual

10. When considering your energy levels over the past 4 weeks, would you say you had a lot of energy?
    - All of the time
    - Most of the time
    - Some of the time
    - A little of the time
    - None of the time

11. Have you felt like your energy levels have changed over the last 4 weeks?
    - Much less energy than usual
    - Somewhat less energy than usual
    - About the same as usual
    - Somewhat more energy than usual
    - Much more energy than usual

12. Over the past 4 weeks, have you experienced low mood?
    - ○ Very often
    - ○ Often
    - ○ Occasionally
    - ○ Infrequently
    - ○ Not at all

13. Over the past 4 weeks, have you felt in control of your health?
    - ○ Not at all
    - ○ Infrequently
    - ○ Occasionally
    - ○ Often
    - ○ Very often

14. During the past 4 weeks, how often have you had trouble sleeping?
    - ○ Not at all
    - ○ Infrequently
    - ○ Occasionally
    - ○ Often
    - ○ Very often

15. Please tell us about how your sleeping experience has been over the past 4 weeks, compared to your usual sleeping experience.
    - ○ Much less sleep than usual
    - ○ Somewhat less sleep than usual
    - ○ About the same sleep as usual
    - ○ Somewhat more sleep than usual
    - ○ Much more sleep than usual

Appendix 3: GHQ Individual Question Results for PP Population*

**GHQ Question Over prior 4 wks**	**Scoring**	**Statistic**	**Visit 1 Day 0**	**Visit 4 Day 30**	**Visit 5 Day 60**	**Visit 6 Day 90**

		# Completed	*36*	*29†*	*33†*	*36*
Question 1: General Health rating	Poor=1, Fair=2, Good=3, Very Good=4, Excellent=5	Mean (SD)	4.0 (0.8)	4.0 (0.8)	4.0 (0.7)	3.7 (1.0)
		Median (min, max)	4.0 (2.0, 5.0)	4.0 (2.0, 5.0)	4.0 (2.0, 5.0)	4.0 (2.0, 5.0)
**Question 2:** Change in General Health	Much worse=1, Some worse=2, Same=3, Some better=4, Much better=5	Mean (SD)	3.2 (0.5)	3.1 (0.4)	3.3 (0.6)	2.8 (0.6)
		Median (min, max)	3.2 (2.2, 4.0)	3.0 (2.0, 4.0)	3.0 (2.0, 5.0)	3.0 (1.0, 4.0)
**Question 3:** Status of GI symptoms	Much worse=1, Some worse=2, Same=3, Some better=4, Much better=5	Mean (SD)	2.9 (0.4)	2.9 (0.9)	2.9 (0.7)	2.9 (0.6)
		Median (min, max)	3.0 (1.0, 4.0)	3.0 (1.0, 5.0)	3.0 (1.0, 4.0)	3.0 (1.0, 4.0)
**Question 4:** Change in Bowel Habits	Much worse=1, Some worse=2, Same=3, Some better=4, Much better=5	Mean (SD)	2.9 (0.3)	2.8 (0.6)	2.9 (0.5)	2.8 (0.5)
		Median (min, max)	3.0 (2.0, 3.0)	3.0 (2.0, 5.0)	3.0 (2.0, 4.0)	3.0 (1.0, 4.0)
**Question 5:** Experience of Brain Fog	Very Often=1, Often=2, Occasion=3, Infrequent=4, None=5	Mean (SD)	4 (0.8)	4.3 (0.7)	4.1 (0.8)	4.1 (0.8)
		Median (min, max)	4.0 (2.0, 5.0)	4.0 (3.0, 5.0)	4.0 (2.0, 5.0)	4.0 (3.0, 5.0)
**Question 6**: Experience of Unhealthy Skin symptoms (rash/itch)	Very Often=1, Often=2, Occasion=3, Infrequent =4, None=5	Mean (SD)	4.5 (0.9)	4.8 (0.5)	4.5 (0.9)	4.6 (0.7)
		Median (min, max)	5.0 (2.0, 5.0)	5.0 (3.0, 5.0)	5.0 (1.0, 5.0)	5.0 (3.0, 5.0)
**Question 7:** Skin Symptom Change Rating	Much worse=1, Some worse=2, Same=3, Some better=4, Much better=5	Mean (SD)	2.9 (0.5)	3.0 (0.4)	3.0 (0.4)	2.9 (0.4)
		Median (min, max)	3.0 (1.0, 4.0)	3.0 (2.0, 4.0)	3.0 (2.0, 4.0)	3.0 (2.0, 4.0)
**Question 8**: Presence of Non-URT	Very Often=1, Often=2, Occasion=3,	Mean (SD)	4.4 (1.1)	4.3 (0.9)	4.2 (1.1)	4.3 (1.1)
		Median (min, max)	5.0 (1.0, 5.0)	5.0 (2.0, 5.0)	5.0 (2.0, 5.0)	5.0 (1.0, 5.0)

(cold/illness) Symptoms	Infrequent=4, None=5					
**Question 9:** Change in URT Symptoms	Much worse=1, Some worse=2, Same=3, Some better=4, Much better=5	Mean (SD)	2.9 (0.5)	2.9 (0.5)	2.8 (0.6)	2.9 (0.5)
		Median (min, max)	3.0 (1.0, 4.0)	3.0 (2.0, 4.0)	3.0 (1.0, 4.0)	3.0 (1.0, 4.0)
**Question 10:** Experienced “a lot” of Energy	None=1, A Little=2, Some=3, Most=4, All=5	Mean (SD)	3.3 (0.8)	3.4 (0.8)	3.4 (0.8)	3.3 (0.8)
		Median (min, max)	3.5 (1.0, 4.0)	4.0 (1.0, 4.0)	4.0 (1.0, 4.0)	3.0 (1.0, 5.0)
**Question 11:** Change in Energy Level	Much Less=1, Some Less=2, Same=3, Some More=4, Much More=5	Mean (SD)	2.8 (0.6)	3.1 (0.5)	3.1 (0.6)	2.8 (0.7)
		Median (min, max)	3.0 (1.0, 4.0)	3.0 (2.0, 4.0)	3.0 (2.0, 4.0)	3.0 (1.0. 4.0)
**Question 12:** Experienced Low Mood	Very Often=1, Often=2, Occasion=3, Infrequent=4, None=5	Mean (SD)	3.8 (1.0)	4.0 (1.0)	3.8 (1.0)	3.9 (0.8)
		Median (min, max)	4.0 (2.0, 5.0)	4.0 (2.0, 5.0)	4.0 (1.0, 5.0)	4.0 (2.0, 5.0)
**Question 13:** Feelings of being in Control of Health	None=1, Infrequent=2, Occasion=3, Often=4, Very Often=5	Mean (SD)	4.2 (0.9)	4.2 (0.8)	3.8 (1.0)	4.0 (0.9)
		Median (min, max)	4.0 (1.0, 5.0)	4.0 (3.0, 5.0)	4.0 (1.0, 5.0)	4.0 (2.0, 5.0)
**Question 14:** Experience of Trouble Sleeping	Very Often=1, Often=2, Occasion=3, Infrequent=4, None=5	Mean (SD)	3.6 (1.0)	3.7 (0.8)	3.9 (0.9)	3.7 (0.7)
		Median (min, max)	3.5 (1.0, 5.0)	4.0 (2.0, 5.0)	4.0 (2.0, 5.0)	4.0 (2.0, 5.0)
**Question 15:** Change in Sleep from Usual Sleeping Pattern	Much Less=1, Some Less=2, Same=3, Some More=4, Much More=5	Mean (SD)	2.7 (0.5)	3.1 (0.5)	2.9 (0.4)	2.7 (0.5)
		Median (min, max)	3.0 (2.0, 4.0)	3.0 (2.0, 5.0)	3.0 (2.0, 4.0)	3.0 (1.0, 3.0)

Abbreviations: d, day; GI, gastrointestinal; max, maximum; min, minimum; SD, standard deviation; URT, upper respiratory tract; wks, weeks

*The data also excludes two individuals consuming supplements that were likely to affect the results as well as two individuals who had baseline timing that were not feasible
